# 

**Published:** 1895-11-23

**Authors:** 


### CONTENTS.

**Philanthropists among the London Poor** - 125
**Tuberculosis in Cattle** - 126
**The Study of Man.**—Practical Anthropology - 127
**The Water Drinker Three Centuries Ago** - 128
**Medical Progress and Hospital Clinics—**
Ischio-Rectal Abscess and Fistula. By E. Percy Paton, M.D. (*continued*) - 131
Progress in Surgery.—Genito-Urinary Surgery (*continued*) - 132
Progress in Obstetrics (*continued*) - 133
Progress in Otology (*continued*) - 134
New Appliances and Things Medical - 136
**Annotations.**—A Statue for Professor Hutley—The New Broom at Walton-on-Naze—Sleep and its Enemies - 129
**Notes and News** - 130
**Editor's Letter Box** - 136
**The Institutional Workshop—**
Hospital Expenditure.—The Commissariat. VII.—The Doctors' Table - 137
Practical Departments - 138
Hospital Meetings - 138
**Scraps and Gleanings** - 139
**The Student of Medicine** - 140

### EXTRA SUPPLEMENT.—THE NURSING MIRROR.

**News from the Nursing World.**—Sad Death of a Private Patient—Birmingham Women's Hospital—An Appeal from Newcastle—Holidays Discouraged—Nursing at Lisburn—A Nurse Out of Work—The Intrusion of Half-Trained Nurses—Cheyne Hospital—Nurses for Ashanti—Male Nurses—Queen's Nurses at Inverness—Short Items - lv

**Elementary Physiology for Nurses.** By C. F. Marshall, late Surgical Registrar, Hospital for Sick Children, Great Ormond Street. XVI.—The Sense of Hearing (*concluded*) - lvii

**Appointments** - lvii

**Where to Go** - lvii

**Presentation** - lix

**The Princess Maud Marriage Present Fund** - lix

**The Penrith Fever Hospital** - lx

**The Half-Trained Nurse** - lviii

**Notes and Queries** - lviii

**Wants and Workers** - lx

**Everybody's Opinion** - lxi

**Novelties for Nurses** - lxii

**For Reading to the Sick** - lxii

